# Deciphering HTLV-1-associated Lung Pathology through Integrated in vitro and Multi-cohort Multi-omics Analysis: Inflammation, Monocyte Recruitment and Differentiation Triggered by HTLV-1-exposed Alveolar Epithelial Cells

**DOI:** 10.21203/rs.3.rs-8051355/v1

**Published:** 2025-11-24

**Authors:** Clément J. F. Heymann, Mieke Gouwy, Robin Hermans, Jean-Claude Twizere, Tatiana Assone, Jorge Casseb, Isaac Racine, Isabelle Cleynen, Edward L. Murphy, Roberta Bruhn, Dominique Schols, Evelien Vanderlinden, Johan Van Weyenbergh

**Affiliations:** KU Leuven Rega Institute for Medical Research.: Katholieke Universiteit Leuven Rega Institute for Medical Research; KU Leuven Rega Institute for Medical Research.: Katholieke Universiteit Leuven Rega Institute for Medical Research; KU Leuven Rega Institute for Medical Research.: Katholieke Universiteit Leuven Rega Institute for Medical Research; University of Liege: Universite de Liege; University of Sao Paulo: Universidade de Sao Paulo; University of Sao Paulo: Universidade de Sao Paulo; KU Leuven: Katholieke Universiteit Leuven; KU Leuven: Katholieke Universiteit Leuven; University of California San Francisco; Vitalant Research Institute; KU Leuven Rega Institute for Medical Research.: Katholieke Universiteit Leuven Rega Institute for Medical Research; KU Leuven Rega Institute for Medical Research.: Katholieke Universiteit Leuven Rega Institute for Medical Research; KU Leuven Rega Institute for Medical Research.: Katholieke Universiteit Leuven Rega Institute for Medical Research

**Keywords:** HTLV-1, transcriptomics, lung, inflammation, monocytes, bronchiectasis, GWAS, interactome

## Abstract

**Background:**

Human T-lymphotropic virus type 1 (HTLV-1) infects up to ten million people worldwide, and causes severe diseases, including adult T-cell leukemia/lymphoma and HTLV-1–associated myelopathy/tropical spastic paraparesis (HAM/TSP). Individuals with HAM/TSP are prone to pulmonary complications (e.g., bronchiectasis). Their bronchoalveolar lavage fluid typically shows increased levels of inflammatory cytokines, chemokines and cell adhesion molecules contributing to chronic inflammation.

**Results:**

This study assessed the impact of HTLV-1 infection on lung inflammation by analyzing the alveolar transcriptome of A549 epithelial cells following exposure to HTLV-1. Co-culture with HTLV-1-infected MT-2 cells caused transcriptomic changes related to viral response, NF-κB activation, and inflammation. RT-qPCR confirmed elevated expression of the chemokine monocyte chemotactic protein-1 (MCP-1/CCL2) and colony stimulating factor 1 (CSF-1) in A549 MT-2 co-cultures. Increased CSF-1 expression was mechanistically linked to NF-κB signaling, using CRISPR/Cas9 RELA knockout. Supernatant from A549 MT-2 co-cultures triggered chemotaxis and macrophage differentiation of THP-1 and primary monocytes. Systems biology analysis revealed enrichment in pathways associated with monocyte infiltration and bronchiectasis. Finally, we validate the *in vivo* relevance of our *in vitro* model through multi-cohort multi-omics analysis combining bulk and single-cell transcriptomics, viral interactomics and multi-ancestry GWAS.

**Conclusions:**

We describe an *in vitro* co-culture model that recapitulates HTLV-1-triggered lung inflammation, through RELA/NF-kB-dependent release of pro-inflammatory cytokines and chemokines resulting in monocyte chemotaxis, activation and differentiation. Integrated multi-omics analysis confirmed the *in vivo* relevance of our *in vitro* model.

## BACKGROUND

Human T-Lymphotropic virus type 1 (HTLV-1) is an enveloped, single-stranded RNA deltaretrovirus affecting up to ten million people worldwide^1,2^. Mainly constrained to endemic areas, HTLV-1 infection is prevalent in the Southwestern part of Japan, sub-Saharan Africa and South America, the Caribbean Islands, and foci in Middle East and Australo-Melanesia Islands^3^. HTLV-1 has been defined as the principal causative agent of two severe diseases, Adult T cell leukemia/lymphoma (ATLL), an aggressive form of T-cell malignancy^4^, and HTLV-1-associated myelopathy/tropical spastic paraparesis (HAM/TSP), an HTLV-1-induced neurologic disorder^5^. HTLV-1 infection can also induce acute inflammation-associated diseases, such as uveitis^6,7^, Hashimoto’s thyroiditis^8^, and Graves’ disease^9,10^. Finally, HTLV-1 carriers, mostly HAM/TSP patients, can exhibit pulmonary complications with the development of T-lymphocyte alveolitis, bronchiolitis or lymphocytic interstitial pneumonia^1,11^.

The first association between HTLV-1 and chronic respiratory disease, i.e. diffuse panbronchiolitis and idiopathic interstitial pneumonia, was published in 1986^12^. This was followed by several reports of T-cell alveolitis and cases of lymphocytosis in broncho-alveolar lavage fluids from HAM/TSP patients^13–15^. Later, the lung was proven to contain one of the highest HTLV-1 proviral loads compared to different organs obtained from the autopsy of an HAM/TSP patient^16^. Currently, all clinical and pathological entities that result from HTLV-1-mediated inflammation of the lung are called HTLV-1-associated pulmonary disease (HAPD)^1^.

HAPD is frequently associated with the emergence of an inflammatory phenotype in the interstitium, airways, or alveoli^1^. Upon infection, respiratory cells produce pro-inflammatory cytokines and chemokines that recruit immune cells to the infected site^17^, where they can either suppress or facilitate viral dissemination. The extravasation of undifferentiated monocytes and peripheral macrophages plays a central role in regulating inflammation and disease progression.

Monocyte trafficking is primarily orchestrated through interactions between CC chemokine receptors (e.g., CCR2, CCR5) expressed on monocytes and their ligands (e.g., CCL2, CCL5) produced by inflamed tissues^17–20^. Once recruited, monocytes can differentiate into macrophages or dendritic cells under the influence of local growth factors, such as macrophage colony-stimulating factor (CSF-1)^21–24^. In the context of HTLV-1 infection, these differentiated monocyte-derived populations may act as viral reservoirs, sustaining viral persistence, and contributing to both immune regulation and tissue immunopathology^25,26^.

One important clinical manifestation of HAPD is bronchiectasis, a chronic lung disorder characterized by the irreversible dilatation and thickening of the walls of the airways^27^. This respiratory disease has been repeatedly associated with HTLV-1 infection, particularly in individuals with HAM/TSP^27–29^. The onset of bronchiectasis is linked to chronic inflammation in the lungs, which fosters the development of a fibrotic microenvironment within the affected tissues. In this setting, monocyte-derived alveolar macrophages have been implicated in the maintenance of pulmonary fibrosis, with their survival and activity supported by CSF-1/CSF-1R signaling pathways^30^.

**Figure 1:**
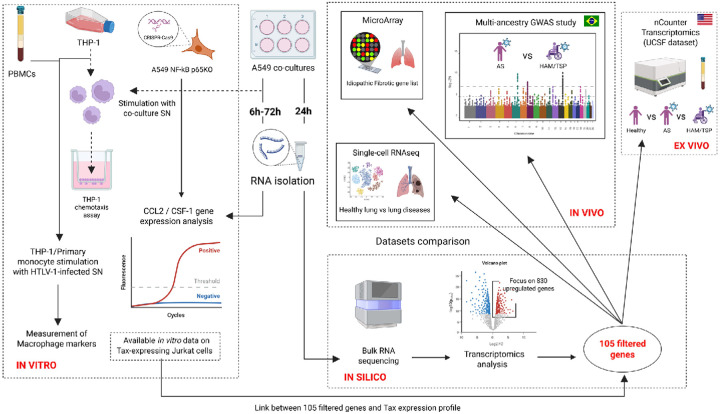
Overview of methodology and cohorts. The study employed an integrative approach to assess the effect of HTLV-1 infection on A549 lung epithelial cells. Differential gene expression analysis was conducted on *in vitro* co-culture samples. Findings were compared with available clinical datasets, highlighting an existing link between the onset of HTLV-1-associated HAM/TSP and idiopathic pulmonary fibrosis.

To better understand HTLV-1-associated inflammatory diseases, particularly HAPD in HAM/TSP patients, this study investigated pro-inflammatory responses triggered by HTLV-1 in A549 alveolar epithelial cells (Fig. 1). HTLV-1 infection in A549 cells was characterized through co-cultures with HTLV-1-infected (MT-4; MT-2) cells, their supernatant (SN) or non-infected (Jurkat) cells for 24 h, followed by bulk RNA sequencing to assess changes in gene expression. HTLV-1-induced inflammation was confirmed by RT-qPCR. The role of the CC chemokine monocyte chemotactic protein-1 (MCP-1/CCL2) and CSF-1 in monocyte recruitment to the lungs was analyzed through kinetic and dose-response studies, with chemotaxis assays, and RT-qPCR was used to evaluate their subsequent differentiation into macrophages. Our findings highlight the role of HTLV-1-induced cytokines in immune cell recruitment and differentiation, which most likely plays a role in viral persistence and immune evasion.

## RESULTS

### 1. exposure to HTLV-1-infected cells or their supernatant alters the transcriptome of A549 alveolar epithelial cells.

**Figure 2:**
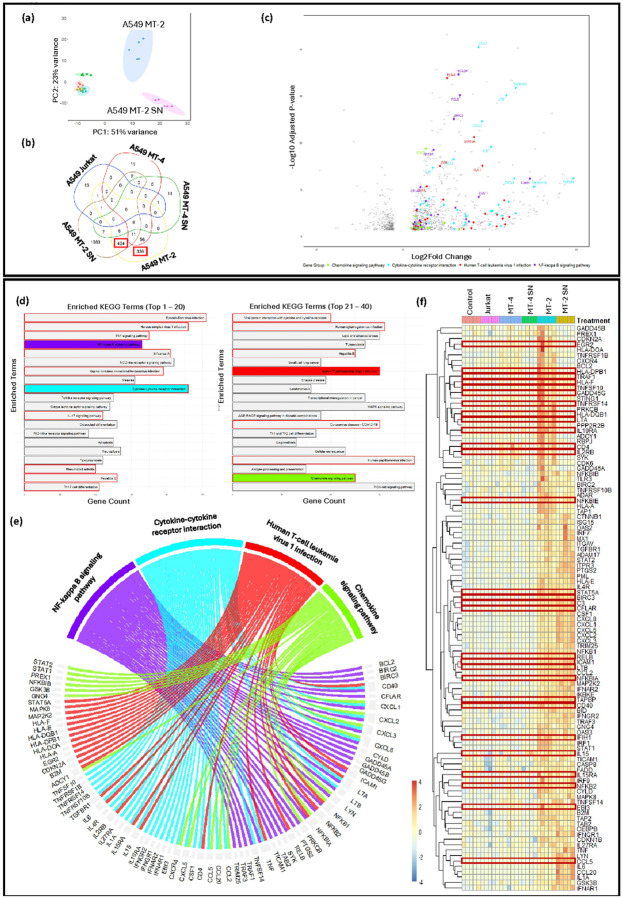
HTLV-1-infected lymphoid cells induce transcriptomic changes in A549 lung epithelial cells. A549 cells (4×10⁵) were co-cultured with Jurkat, MT-4 (or SN), or MT-2 (or SN) cells at a 1:1 ratio for 24 h (n = 4–6), and RNA was extracted for sequencing. **(a)** PCA shows distinct clustering of A549 cells (orange), and A549 cells co-cultured with MT-2 cells (blue), MT-2 SN (purple), MT-4 cells (green), MT-4 SN (turquoise) and Jurkat cells (red). **(b)** Venn diagram highlights unique and overlapping upregulated DEGs, with MT-2 cells and MT-2 SN showing distinct profiles. **(c)** Volcano plot displays DEGs in A549 MT-2 vs A549 Jurkat controls. **(d)** KEGG over-representation analysis of 830 MT-2–specific DEGs. Top 40 enriched pathways are shown, with selected pathways highlighted. **(e)** Chord diagram maps 105 clinically relevant DEGs shared across 4 of 18 selected KEGG pathways. **(f)** Heatmap shows fold changes of the 105 filtered genes across all co-culture conditions. Genes highlighted in red were shown to be positively regulated by HTLV-1 Tax^33^.

The lung constitutes one of several organs likely to be affected by HTLV-1-mediated inflammation. To evaluate the impact of HTLV-1 exposure on gene expression in lung epithelial cells, bulk RNA sequencing was performed on A549 cells co-cultured with MT-2, MT-4 or Jurkat cells for 24h (Supplementary Tables 2–6). In parallel, A549 cells were exposed to supernatant (SN) from MT-2 or MT-4 cell cultures to determine the gene expression differences driven by factors like cytokines in the SN of HTLV-1-infected cells. Principal Component Analysis (PCA) revealed treatment-specific clustering, with clear distinction between the different co-culture conditions (Figure 2a). Sequencing reads were aligned to both the human genome and HTLV-1 reference genome J02029.1. Notably, alignment to the HTLV-1 genome highlighted the presence of viral reads (e.g., reads aligning to HTLV-1 *Gag* and *Pol* sequences) in A549 cells co-cultured with MT-2 cells (Table 2). In contrast, only a small number of HTLV-1-mapped reads were detected in the A549 MT-4 co-cultures, which aligned with RT-qPCR results (Supplementary Figure 1c). Reads mapped to the human genome were subsequently used for differential gene expression analysis (Figure 2, Supplementary Figure 1).

DESeq2 software was used to compare gene expression in A549 cells co-cultured with HTLV-1–infected cells, their SN, or non-infected cells, across an average of 14,400 genes detectable above background (Table 3). To confirm that the RNA originated from A549 cells, the DEGs were cross-referenced with known alveolar epithelial markers (Supplementary Figure 2). Cluster analysis of these markers across the different samples showed no noteworthy differences between treatment groups, indicating overall sample homogeneity (Supplementary Figure 2). In line with the PCA analysis, both Venn diagrams and volcano plot confirmed a distinct transcriptional profile observed in A549 cells co-cultured with MT-2 cells, compared to both A549 co-cultures with Jurkat or MT-4 cells (Figure 2b, 2c, Supplementary Figure 1).

HTLV-1 spreads primarily via cell-to-cell contact. While co-culture systems accurately model this process, it often results in complex mixtures of donor and target cell materials, complicating downstream analyses. Residual HTLV-1-infected cells may adhere to A549 cells, obscuring epithelial-specific transcriptomic changes. To address this, transcriptome deconvolution was performed using CIBERSORTx^31^ to quantify potential contamination by MT-2 or MT-4 transcripts, identified through digital transcriptomics (Vanderlinden et al., unpublished data) (Supplementary Figure 1d). As shown in Supplementary Figure 1d, no significant increase in MT-2 or MT-4–specific transcripts was observed across all experimental conditions.

While only 80–103 (0.6–0.7%) DEGs were identified (padj <0.05 after stringent FDR correction) in Jurkat and MT-4 conditions, 1304 (8.5%) and 2956 (19.8%) genes were significant in A549 MT-2 and A549 MT-2 SN co-cultures, respectively (Table 3). Most DEGs in A549 MT-2 or MT-2 SN conditions were unique, with 336 (37%) and 1083 (68%) of upregulated genes exclusive to each treatment, respectively (Figure 2b). In contrast, A549 co-cultured with MT-4 cells or MT-4 SN displayed similar transcriptomic profiles to A549 Jurkat control (Figure 2a, 2b, Table 3). Interestingly, SN exposure induced stronger gene downregulation, with 45.7% of downregulated DEGs in A549 cells exposed to MT-2 SN compared to 30.6% in MT-2 co-culture settings (Table 3). Log2 fold changes and adjusted p-values of the 20 most significant DEGs (padj < 0.05) per condition are summarized in Supplementary Table 9. For downstream analysis, a focus was given to the 830 genes significantly upregulated in A549 cells co-cultured with MT-2 cells (Figure 2b, red boxes).

A systems biology analysis was performed on the 830 selected DEGs to identify key biological pathways influenced by exposure of the A549 cells to HTLV-1 (Supplementary Table 10). The 830 DEGs were mainly linked to various viral infections, as shown by the significant enrichment of KEGG terms, such as **“Human T-cell leukemia virus 1 infection,” “Epstein-Barr virus infection,”** and **“Hepatitis B/C infection”** (Figure 2d), all linked to cancer and/or (neuro)inflammation. Complementary Gene Ontology (GO) enrichment analysis confirmed enrichment in terms such as **“Defense Response to Virus”** and **“Viral Process”** (Supplementary Figure 3). Moreover, KEGG over-representation analysis revealed strong activation of inflammatory pathways, including **“TNF signaling pathway”, “NF-kappa B signaling pathway”** and **“IL-17 signaling pathway”** (Figure 2d). These observations were consistent with elevated pro-inflammatory cytokine levels measured in A549 MT-2 co-cultures (Supplementary Figure 5a-5d). In addition to inducing inflammation, HTLV-1 exposure also activated both innate and adaptive immune responses in A549 cells, as revealed by enrichment in pathways, such as **“Toll-like receptor signaling”** and **“Cytokine-cytokine receptor interactions”** (Figure 2d). Of note, the upregulation of the **“Chemokine signaling pathway”** suggested the enhanced interplay between HTLV-1-exposed A549 cells and nearby immune cells during HTLV-1 infection (Figure 2d). This finding was supported by GO analysis, which revealed enrichment of pathways related to **“Leukocyte chemotaxis”, “Monocyte differentiation”** or **“Macrophage activation”,** indicating immune cell engagement at sites of HTLV-1 exposure (Supplementary Figure 4, Supplementary Table 11).

**Figure 3:**
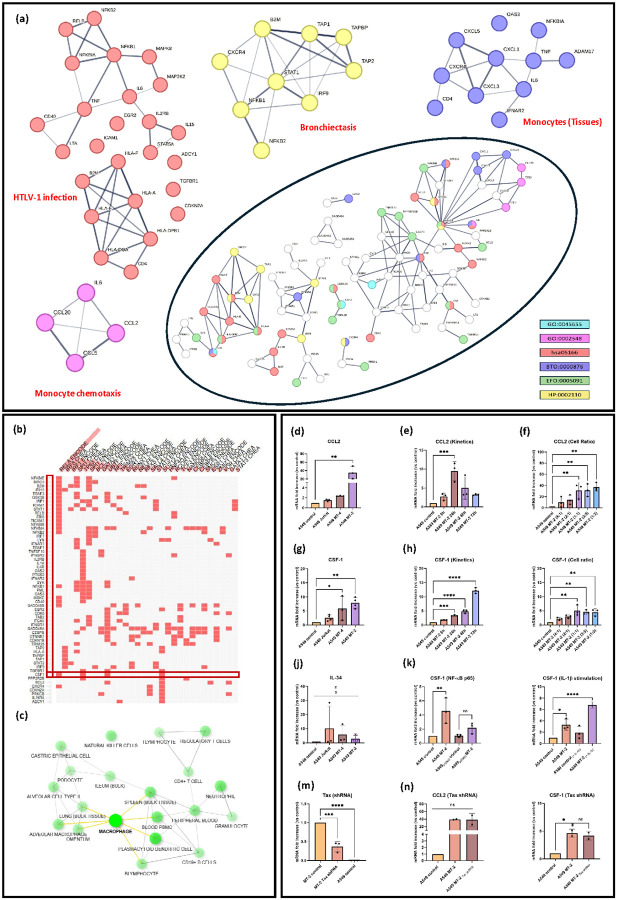
HTLV-1 infection promotes pro-inflammatory signaling and immune activation in lung epithelial cells. Among 830 upregulated genes in A549 MT-2 cells, 105 were selected based on overlap with ≥2 of 18 enriched KEGG pathways. **(a)** PPI network analysis grouped the associated proteins into 5 biological processes, highlighting enrichment in HTLV-1 pathway (**hsa05166,** red), monocyte differentiation (**GO:0045655,** cyan), monocyte recruitment (**GO:0002548,** magenta), tissue-resident monocyte activity (**BTO:0000876,** dark blue), and bronchiectasis-related inflammation (**HP:0002110,** yellow). **(b, c)**Transcription factors (TFs) regulating these proteins were identified through enrichment analysis. (**d–i**) RT-qPCR showed cell ratio- and time-dependent increases in CCL2 and CSF-1 expression following MT-2 co-culture. (**j–l**)In p65KO A549 cells, CSF-1 upregulation was suppressed, implicating canonical NF-κB signaling. **(m–o)** Tax knockdown in MT-2 cells did not significantly alter CCL2 or CSF-1 expression in A549 cells. Statistical analysis by one-way ANOVA with Tukey post hoc test; *p < 0.05, **p < 0.01, ***p < 0.001, ****p < 0.0001.

To further evaluate the impact of HTLV-1 infection on alveolar epithelial cells, the 830 upregulated DEGs from A549 MT-2 co-cultures (Figure 2b, 2f) were filtered based on 18 KEGG pathways clinically relevant to HTLV-1 infection (i.e., pathways associated with oncogenic, (neuro)inflammatory, or respiratory viral infections) (Figure 2d, Supplementary Table 10). This biological filtering process yielded 105 of the 830 upregulated DEGs (Figure 2f). To illustrate their distribution across a subset of selected pathways, a circus plot was generated, providing a global overview of the pathway-gene relationships (Figure 2e). These genes were subsequently used to construct a PPI network, which highlighted hub proteins essential for crucial cellular processes and bottleneck proteins known to regulate multiple pathways simultaneously(Figure 3a). Notably, 25 of these proteins were significantly enriched in the KEGG pathway **“Human T-cell leukemia virus type 1 infection”,** supporting the relevance of the experimental model. STRING analysis further highlighted enrichment in terms such as **“Tissue monocytes”** and **“Bronchiectasis”,** aligning with KEGG results, previously reported clinical data, and recent multi-omics findings (Figure 3a)^28,29,32^.

The role of monocyte recruitment in virus-induced pulmonary inflammation was further supported by the enrichment of the GO term **“Monocyte chemotaxis”** in the PPI network, driven by key chemokines such as CCL2, CCL5, CCL20, and the cytokine IL-6 (Figure 3a). Thus, upon recruitment to the lungs, monocytes might undergo differentiation into inflammatory or profibrotic macrophages, as indicated by enrichment of the term **“Regulation of monocyte differentiation”** and increased expression of growth factors, like CSF-1 (Figures 2f). Activation of the CSF-1R signaling axis was further supported by enhanced presence of JAK/STAT pathway components, including STAT1, STAT2, and STAT5A. Notably, STAT5A is known to promote expression of the anti-apoptotic gene BCL2, which was also present among the filtered KEGG gene list, suggesting a potential mechanism for increased cell survival during infection (Figures 2f, 3a).

### 2. Exposure to HTLV-1 drives the development of a pro-inflammatory alveolar microenvironment and leads to recruitment of immune cells to the lungs.

Given the respiratory complications observed in both HAM/TSP patients and individuals living with HTLV-1, this study assessed the potential of HTLV-1 to induce a pro-inflammatory microenvironment in epithelial cells. To this end, A549 cells were co-cultured for 48 hours with HTLV-1–infected MT-2 or MT-4 cells, or with uninfected Jurkat cells, and mRNA levels of key pro-inflammatory cytokines (*IL-6, CXCL8, IL-1β, TNF-α*) were measured(Supplementary Figure 5) In addition, mRNA levels of chemokines and growth factors, including *CCL2, CSF-1,* and *IL-34* were measured in epithelial cells(Figure 3d–3l).

CSF-1, a key regulator of monocyte proliferation and differentiation, was significantly upregulated in A549 cells co-cultured with MT-2 cells (Figure 3g). By contrast, levels of IL-34, another cytokine that binds CSF-1R, remained unchanged upon co-culture with HTLV-1–infected cells across the different conditions, confirming the CSF-1–specific upregulation (Figure 3j). CSF-1 expression was the highest at an A549:MT-2 cell ratio of 1:1 (Figure 3i), and increased over time, reaching a 13-fold rise at 72 hours (Figure 3h).

Upstream transcription factor (TF) enrichment analysis, using the ENCODE database, was performed on the 105 KEGG-filtered genes to identify principal transcriptional regulators. This systems-level approach revealed several NF-κB-related TFs, with RELA (NF-κB p65) emerging as a prominent candidate for the selected genes (Figure 3b). Complementary analysis using the ARCHS4 Tissue database further indicated that these genes are predominantly regulated by TFs active in macrophages, including alveolar macrophages (Figure 3c). To decipher whether *CSF-1* upregulation was indeed driven by NF-κB activation, an A549 RELA (NF-κB p65) knockout cell line was generated using CRISPR-Cas9. When co-cultured with MT-2, these knockout cells did not exhibit significant *CSF-1* induction (Figure 3k). In contrast, IL-1β stimulation enhanced *CSF-1* expression in A549 cells (Figure 3l). The contribution of HTLV-1 *Tax* was also evaluated but showed no effect, as CSF-1 expression remained unchanged when A549 cells were stimulated with MT-2 Tax shRNA cells (Figures 2f, 3o). This finding corroborates a recent transcriptomics study performed in Jurkat cells expressing *Tax,* where *Tax* was shown not to regulate CSF-1 (Figure 2f)^33^.

CCL2 is a chemokine that plays a key role in the immune response by acting as a chemoattractant, primarily recruiting monocytes and other immune cells to sites of inflammation or tissue injury. In line with RNA-seq data (Figure 2f, Supplementary Table 9), A549 cells co-cultured with MT-2 cells showed a significant increase in CCL2 levels compared with A549 cells alone (Figure 3d). CCL2 expression was maximal at 24 h incubation, and increased with higher MT-2 cell number, indicating a cell ratio- and time-dependent regulation (Figure 3e, 3f). Together, these findings indicate that HTLV-1 exposure promotes the expression of both CCL2 and CSF-1, key mediators of monocyte recruitment to the lung epithelium (Figure 3d–l).

**Figure 4:**
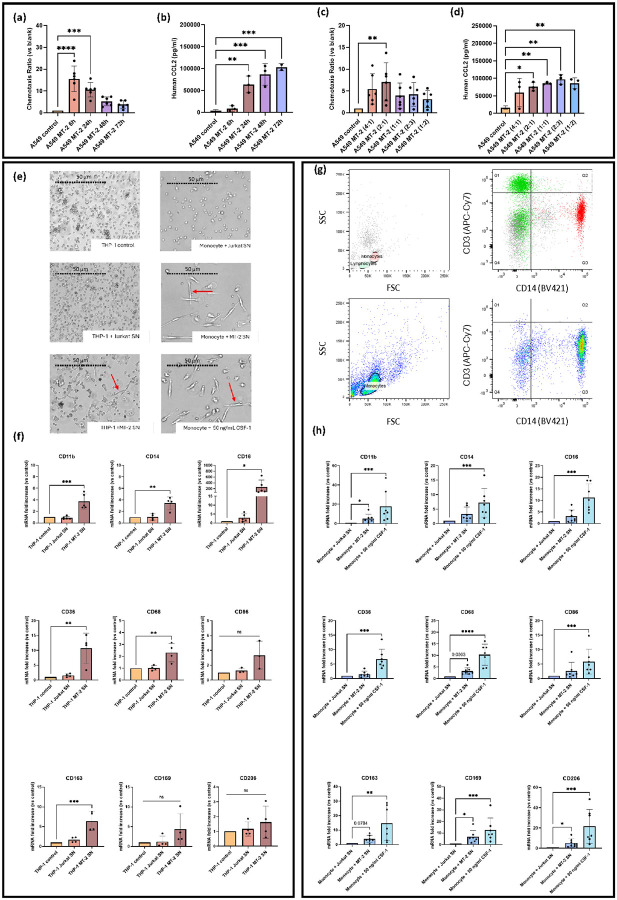
HTLV-1–induced factors drive monocyte recruitment and macrophage differentiation. **(a–d)** A549 cells were co-cultured with MT-2 cells at different ratios and for varying durations. **(a, c)** SN from these cultures was used in THP-1 chemotaxis assays. **(b, d)** ELISA confirmed CCL2 levels, with peak THP-1 migration observed for SN harvested at 6h, despite lower CCL2 levels. **(e)** SN from A549–MT-2 co-cultures induced elongation and flattening in THP-1 and primary monocytes, resembling CSF-1–treated cells. **(f)** RT-qPCR after 5-day THP-1 culture in MT-2 SN showed upregulation of macrophage markers, indicating differentiation. **(g)** Primary CD14^+^ monocytes were isolated from healthy donors via density gradient and magnetic sorting, with purity validated by flow cytometry. **(h)** CD14^+^ monocytes (n = 7) were cultured with Jurkat SN, MT-2 SN, or CSF-1. RT-qPCR confirmed macrophage marker induction, validating THP-1 findings. Statistical significance: one-way ANOVA with Tukey test for THP-1 RT-qPCR; *p < 0.05, **p < 0.01, ***p < 0.001, ****p < 0.0001; Kruskal-Wallis used for primary monocytes RT-qPCR.

To evaluate the role of HTLV-1 in monocyte recruitment, chemotaxis assays were performed using THP-1 monocytic cells exposed to increasing concentrations of CCL2 (1–30 ng/mL). CCL2 clearly induced cell migration at concentrations ≥10 ng/mL (Table 4). In parallel, assays using SN from A549–MT-2 co-cultures revealed a time-dependent increase in cell migration (Figure 4a), which correlated with increased CCL2 levels in the SN (Figure 4b). Interestingly, THP-1 migration was highest for SN harvested at 6 hours (CI: 16 + 0.7, n=9) and declined substantially by 48 hours (CI: 5.8 ± 0.7, n=9) (Figure 4a, Table 4), indicating that migration peaked early on at suboptimal concentrations of CCL2 (±10 ng/mL) (Table 4). Similarly, increasing the number of MT-2 cells in co-culture led to higher CCL2 concentrations in the SN (Figure 4d), while chemotaxis peaked at an A549:MT-2 ratio of 2:1 (Figure 4c). As CCL2 levels continued to rise, THP-1 chemotactic responsiveness declined (Figure 4c, 4d, Table 4), suggesting that excessive chemokine concentrations may desensitize monocytes to chemotactic gradients.

Although CD4^+^ T cells are the primary target of HTLV-1 infection, monocytes are also potential candidates. To explore the effects of cell–cell contact, and soluble factors secreted by HTLV-1-infected cells on monocyte recruitment and differentiation, THP-1 monocytic cells were cultured for six days in either standard medium or medium conditioned by Jurkat or MT-2 cell cultures (Figure 4e, 4f). THP-1 cells exhibited notable morphological changes when cultured with MT-2 SN, adopting an elongated shape, and becoming adherent (Figure 4e). To further characterize these polarized cells, RNA was extracted from various THP-1 co-cultures, and RT-qPCR was performed to assess the expression levels of different macrophage surface markers (Figure 4f). THP-1 cells exposed to MT-2 SN showed increased mRNA levels of CD11b, CD14, and CD16, 3 markers commonly associated with myeloid and monocyte lineage. Similarly, elevated expressions of macrophage-specific markers CD36, CD68 and CD163 were measured, indicating a shift toward a macrophage-like phenotype (Figure 4f).

To confirm the clinical relevance of the *in vitro* findings, we validated the expression of key markers identified in THP-1 cells (Figure 4f), using primary monocytes isolated from PBMCs of healthy donors (Figure 4h). Monocytes were purified by negative selection and confirmed by multicolor flow cytometry using CD3 (APC-Cy7) and CD14 (BV421) staining (Figure 4g). The cells were then cultured in conditioned media or stimulated with 50 ng/mL CSF-1 to induce macrophage differentiation. Similar to THP-1 results, primary monocytes exposed to MT-2 SN or CSF-1 underwent notable morphological changes (Figure 4e). Both treatments increased CD11b expression (Figure 4e). While CSF-1 stimulation significantly upregulated all tested macrophage markers (CD36, CD68, CD86, CD163, CD169, and CD206), MT-2 SN specifically induced significant increases in CD169 and CD206 only (Figure 4h).

### 3. Transcriptomic analysis of alveolar epithelial cells identifies multi-omics markers of HTLV-1-associated disease and idiopathic pulmonary fibrosis.

**Figure 5:**
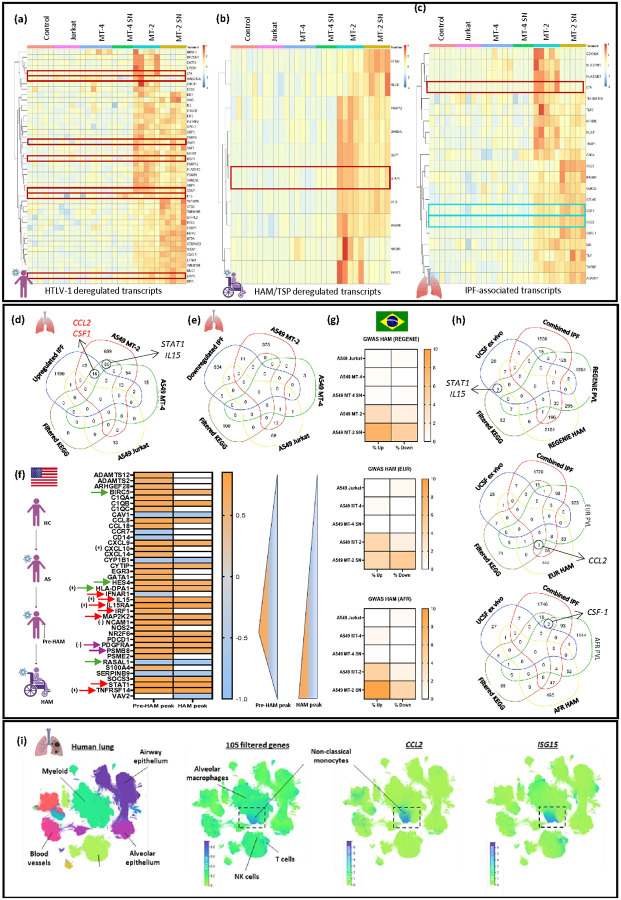
Transcriptomic overlap between HTLV-1–induced lung inflammation, HAM/TSP, and IPF signatures. **(a–b)** DEGs (n = 830) from A549 MT-2 co-cultures were compared to HTLV-1–deregulated PBMC transcripts reported by Tattermusch *et al*. (2012), including 80 HAM/TSP-specific genes. Fold changes are shown for overlapping transcripts. Genes highlighted in red are the genes that are also represented in the filtered KEGG gene list. **(c)** A curated list of IPF-associated genes was compiled from five GEO datasets (**GSE32537, GSE47460, GSE53845, GSE70866, GSE110147**). Genes significantly deregulated in at least three datasets were cross-referenced with the 105 KEGG-filtered DEGs from A549–MT-2. The heatmap highlights shared genes, with those in red representing overlap with the HTLV-1–deregulated gene list (see 5a), and those in cyan denoting the two genes selected as focal points for the *in vitro* experiments (see Figure 3). **(d,e)** Venn diagrams show overlap between curated IPF gene sets and DEGs from various A549 co-cultures. **(f)**UCSF *ex vivo* transcriptomic data identified genes associated with HTLV-1 clinical status and/or Disease Burst (Purple: IPF downregulated; Green: IPF upregulated; Red: Filtered KEGG gene list; (−/+): Genes negatively or positively regulated by HTLV-1 Tax). Statistical significance: padj < 0.05. **(g)**Heatmaps illustrate overlap percentages between GWAS datasets and A549-derived DEGs. **(h)** Venn diagrams summarize intersections across UCSF gene sets, IPF-up/downregulated genes, KEGG-filtered genes, and GWAS hits. **(i)**UMAPs from single-cell data show expression profiles of overlapping genes across key cell types.

In the context of HAM/TSP, genome-wide transcriptome analysis of whole blood samples has enabled the identification of disease-specific biomarkers, supporting the development of targeted diagnostics^34,35^. In this study, DEGs measured from the different A549 co-culture conditions (Supplementary Tables 2–6) were compared to previously published datasets^32,34,36,37^ using systems biology analysis to evaluate their concordance with known HTLV-1-associated biomarkers (Figure 5a, 5b). Specifically, significantly upregulated DEGs in A549 MT-2 co-cultures (Figure 2b, red box) were compared with whole blood transcriptome signatures from Tattermusch *et al*.^34^ (**GSE29312**), who reported 542 HTLV-1-deregulated transcripts, including 80 specifically linked to HAM/TSP.

Comparative analysis revealed 44 overlapping genes with the HTLV-1 signature profile and 10 with the HAM/TSP-specific subset (Figure 5a). Notably, 7 of the 105 genes from our KEGG pathway enrichment list were found among the 44 shared genes, including *GADD45A, LTA,* interferon-regulated genes *OAS3* and *ISG15,* and immune regulators involved in monocyte recruitment and differentiation *STAT1, IL15,* and *CXCL5* (Figure 5a). Of interest, *STAT1* was identified as a key HAM/TSP biomarker both *in silico* and *in vivo*^38,39^, highlighting its potential role in disease pathogenesis (Figure 5b).

Beyond comparisons with general HTLV-1 and HAM/TSP biomarkers (Figure 5a, 5b), the same DEGs were cross-referenced with a recent multi-ancestry GWAS^32^ for both HAM/TSP and proviral load (PVL) (Figure 5g). A549 cells co-cultured with MT-2 or exposed to MT-2 SN exhibited transcriptomic profiles that closely aligned with gene expression patterns observed in the different HAM/TSP GWAS cohorts (Figure 5g). Notably, 4–10% of deregulated genes under both conditions overlapped with GWAS findings, highlighting a strong association with both HAM/TSP diagnosis and elevated HTLV-1 proviral load (PVL) (Figure 5g, Supplementary Figure 6). Key overlapping genes included regulators critical for monocyte recruitment and macrophage differentiation, such as *CCL2* in the European cohort and *CSF-1* in the African cohort (Figure 5h).

HTLV-1-associated lung pathology may lead to bronchiectasis, an inflammatory condition linked to IPF development (Figure 3a). To explore the potential role of HTLV-1 in promoting IPF-like changes in lung epithelial cells, a cross-analysis of publicly available transcriptomic datasets was performed, incorporating samples from confirmed IPF patients and healthy donors (**GSE32537**^40^**, GSE47460**^41–45^**, GSE53845**^46^**, GSE70866**^47^**, GSE110147**^48^) (Figure 5c–5e). An IPF gene list was compiled through consensus analysis across these datasets, retaining genes that were consistently deregulated in IPF samples in at least three datasets. Genes that appeared in both up- and downregulated sets across datasets were excluded from the final list to ensure robustness. Among our 105 filtered KEGG genes (Figure 2f), 21 overlapped with the IPF-upregulated gene set (Figure 5c), including immune-related chemokines and growth factors such as *CCL2, CXCL1, CCL5,* and *CSF-1. TNF-α,* a key inflammatory regulator, was also commonly upregulated, suggesting a mechanistic link between HTLV-1-induced immune modulation and fibrotic remodeling in pulmonary tissue (Figure 5c).

Deregulated genes from the different GWAS cohorts (Figure 5g) were compared to the IPF gene list, focusing on significantly upregulated genes (Figure 5h). These were cross-referenced with our filtered KEGG gene list (Figure 2f) and an additional *ex vivo* UCSF dataset^36,37^ using a different platform (nCounter, Nanostring), correlating transcriptomic profiles of HAM/TSP patients to their disease status and PVL. Venn diagram analyses revealed significant overlap among the filtered KEGG gene list, the IPF gene set, and the *ex vivo* UCSF dataset (Figure 5h). Notably key immune-related regulators, including *STAT1* or *IL-15*, were found in both the filtered KEGG gene list and the *ex vivo* UCSF dataset. These genes play a central role in interferon signaling, monocyte activation, and antiviral defense (Figure 5h). Consistently, these results supported the protein-protein interaction (PPI) network shown in Figure 3a, where several of these regulators appeared as central nodes in pathways enriched across both KEGG and GWAS analyses (e.g., STAT1, TNF-α). Together, this convergence of transcriptomic and genomic evidence highlights the potential involvement of these regulators in HAM/TSP pathogenesis and progression.

The 105 filtered KEGG genes were further analyzed using single-cell RNAseq data from the integrated Human Lung Cell Atlas (HLCA)^49^ (Figure 5i). The HLCA is an open-access resource comprising over 2 million respiratory tract cells collected from 486 individuals, encompassing 49 distinct datasets. Its core includes data from healthy lung tissue, which can be directly compared to samples from individuals with various lung diseases. Analysis showed that expression of the 105-gene signature (Figure 2f) was elevated in multiple inflammatory lung conditions, such as pulmonary fibrosis, COPD, and COVID-19 (Supplementary Figure 7). Additionally, cross-comparison identified a distinct myeloid cell subset characterized by high CCL2 expression, which aligned with the known HAM/TSP type I interferon (IFN) gene signature (Figure 5i and Supplementary Figure 7). Notably, this specific myeloid subset was characterized by a strong correlation between *CCL2* and *ISG15/CXCL10* expression (Supplementary Figure 7b-7c), two IFN-regulated genes commonly upregulated in HAM/TSP patients, of which CXCL10 has been validated as a bona fide biomarker for clinical evolution in HAM/TSP^50–53^.

## DISCUSSION

While HTLV-1 tropism for lung tissues is well established^1,12,15,54^, its interaction with non-lymphoid cells, particularly epithelial cells, remains unclear. Previous *in vitro* studies demonstrated that alveolar epithelial cells could in fact harbor HTLV-1, as shown by the detection of proviral DNA and viral proteins in A549 cells, following exposure to HTLV-1-infected cells^17^. In this study, we assessed the effects of HTLV-1 exposure on A549 cells, using a multi-omics approach. To investigate the cellular mechanisms underlying the chronic inflammation characteristic of HAPD, transcriptomic profiling was performed on A549 cells following co-cultures with HTLV-1-infected MT-2 or MT-4 cells, or non-infected Jurkat cells (Supplementary Tables 2–6). In parallel, A549 cells were also stimulated with MT-2 or MT-4 SN to determine the impact of HTLV-1-associated soluble factors on gene expression. DEGs analysis revealed stimulus-specific transcriptional responses, with a substantially higher number of deregulated genes detected in A549 cells exposed to MT-2 cells or their SN (Fig. 2b, Supplementary Fig. 1). Enrichment analysis of these DEGs identified pathways associated with antiviral defense, cytokine signaling, and NF-κB activation, which drew the selection of 105 candidate genes (HTLV-1 signature) for downstream analysis (Fig. 2f).

HTLV-1-induced inflammation is characterized by an enhanced immune response within infected tissues. In the lung, this includes elevated numbers of T lymphocytes in bronchoalveolar lavage fluid (BALF)^55–58^ and high HTLV-1 PVL^59^, both of which contribute to chronic inflammatory responses. Beyond acting as physical barriers, lung epithelial cells actively participate in immune surveillance, by producing cytokines and chemokines that may influence HAPD progression. In response to HTLV-1 exposure, A549 cells mounted a robust antiviral response, marked by the upregulation of interferon-stimulated genes (*TNFSF14, ISG15, OAS3*) and interferon receptor genes (*IFNAR1, IFNGR1, IFNGR2*) (Fig. 2f). A key node in this interferon-driven response is STAT1, a central mediator of inflammatory signaling and antiviral responses^60^. By transducing interferon (IFN) signals^60^, STAT1 regulates the expression of a broad array of antiviral and pro-inflammatory genes, thereby shaping the host immune response to HTLV-1. Notably, STAT1 dysregulation has been previously reported in HAM/TSP patients. Indeed, Tattermusch *et al*. (2012) measured elevated STAT1 protein levels in these patients and linked this to type I IFN signature^34^. Accordingly, our curated KEGG HTLV-1 gene signature revealed a STAT1 upregulation across multiple datasets, both *in silico* (Fig. 2f) and *in vivo* with patient-derived samples (Fig. 5a, 5b, 5h). This parallel suggests that STAT1-driven inflammatory pathways observed in A549 cells may mirror mechanisms contributing to HTLV-1-associated lung pathology *in vivo*.

In addition to antiviral genes, A549 co-culture with MT-2 cells increased expression of pro-inflammatory cytokines (*TNF-α, IL-6, CXCL8,* and *IL-1A*), which reflects the heightened inflammatory state observed in HTLV-1-exposed A549 cells (Fig. 2f and Supplementary Fig. 5). These findings align with a previous *in vitro* study reporting increased production of pro-inflammatory cytokines and chemokines in HTLV-1-infected A549 cells^17^. Remarkably, elevated TNF-α has been associated with clinical worsening in HAM/TSP, while the systemic increase in IL-6 has been linked to inflammaging, a common phenomenon observed in HAM/TSP patients^36^.

The observed HAPD-induced pro-inflammatory response seems, at least partially, mediated by NF-κB signaling. Indeed, HTLV-1-exposed A549 cells exhibited increased expression of NF-κB-related genes (*NFKB1, NFKB2, RELA*) (Fig. 2f), consistent with the activation of pro-inflammatory and antiviral pathways. Among the NF-κB–regulated genes, *IL-15* was particularly notable due to its strong association with both epithelial immune signaling^61^ and the Th1-biased inflammatory response^62^ observed in HAM/TSP patients. *IL-15* expression is *Tax*-dependent^63^ and tightly regulated by NF-κB (Fig. 2h). IL-15 can be found at elevated levels in PBMCs of HAM/TSP patients. Blocking *IL-15* expression can reduce PBMC proliferation^64^, underscoring its role in disease progression. In this study, the consistent upregulation of *IL-15* across transcriptomics (Fig. 2f, 5a and 5f) and GWAS datasets (Fig. 5g), highlights its potential as both a biomarker and therapeutic target in HTLV-1–associated pulmonary inflammation.

Epithelial-driven pro-inflammatory signaling amplifies cytokines and chemokines production, which favors the recruitment of immune cells (e.g., monocytes) to the lungs. Macrophages are among the most abundant immune cells in the respiratory tract^65^ and are essential for antiviral defense, controlling inflammation^66^, and preserving tissue homeostasis^67,68^. Their versatility allows them to adopt either pro-inflammatory or anti-inflammatory phenotypes, depending on environmental cues^69^. During viral infection, monocytes migrate to inflamed sites in response to chemotactic signals^18^ and differentiate into macrophages, which can either promote pathogen clearance or support tissue repair^19^.

In the present study, co-culturing A549 cells with HTLV-1–infected MT-2 cells induced a strong pro-inflammatory chemokine response (*CCL2, CCL5, CCL20*) and increased production of the local growth factor *CSF-1* (Fig. 2h). Chemotaxis assays with THP-1 cells confirmed a cell ratio- and time-dependent increase in monocyte migration toward A549 MT-2 SN (Fig. 4a, 4c). THP-1-induced cell migration followed a typical Gaussian distribution, with an optimal chemokine concentration eliciting maximal migration. As of 24 h, the concentration of CCL2 present in the SN was likely supra-optimal, resulting in reduced THP-1 cell migration (Fig. 4a). Comparison of different cell ratios also showed reduced chemotaxis at a CCL2 concentration of 100 ng/mL, which is most likely caused by receptor desensitization (Fig. 4c). Beyond promoting monocyte recruitment, factors secreted into A549 MT-2 SN may also influence myeloid cell fate. Indeed, exposure of THP-1 cells or primary monocytes to MT-2 SN promoted their differentiation into macrophages (Fig. 4e, 4h), an effect that correlated with the increased mRNA levels of CCL2 and CSF-1 observed in the A549 MT-2 co-culture (Fig. 2d, 2g). Using a CRISPR/Cas9 RELA knockout cell line, we confirmed that NF-κB regulates CSF-1 expression in A549 cells in response to MT-2 co-culture, which further highlights the pivotal effect of HTLV-1 on NF-κB signaling.

In the context of HAPD, the presence of monocytes and differentiated macrophages in inflamed tissues can serve as a prognostic biomarker for pulmonary fibrosis^70,71^. Indeed, elevated monocyte counts in the lung were previously associated with an increased risk of IPF progression, hospitalization or death^70,72,73^. On the other end, lung fibrogenesis has been shown to decrease significantly following depletion of circulating monocytes or when macrophage recruitment to the lung is blocked after injury in mouse models^74^. The increased recruitment of monocytes during IPF development was recently linked to age-associated changes. Farhat *et al*. (2025) demonstrated that an aged hematopoietic system can enhance the risk of lung fibrosis in young mice^75^. This effect was associated with an increased influx of monocytes, which gave rise to profibrotic macrophages in lung tissue^75^. In this study, STRING analysis of our curated KEGG gene list identified different hub proteins, including tissue monocyte markers as well as bronchiectasis-associated proteins (Fig. 3). Together, these findings underscore the capacity of HTLV-1–exposed epithelial cells to influence, not only monocyte recruitment but also the functional activation of myeloid cells in the lung and its contribution to IPF development. All these results aligned with findings from a recently published study, in which the authors confirmed the role of secreted factors of HTLV-1-infected cells in monocyte activation and differentiation^25^.

To explore the *in vivo* relevance of our *in silico* and *in vitro* findings, we refined an HTLV-1 infection signature by cross-referencing our expression data with published whole blood transcriptomic profiles from HAM/TSP patients^34^ (Fig. 5a, 5b). Genes upregulated in A549 MT-2 co-cultures also showed a strong overlap with a curated IPF gene list (Fig. 5c–5e) and multi-ancestry GWAS data (Fig. 5g). In a recent preprint, Assone *et al*. used systems biology analyses of novel and publicly available data comprising (epi)genomics, transcriptomics, metabolomics and proteomics of multi-ancestry cohorts from a total of > 2500 people living with HTLV-1 from 5 countries (Brazil, Peru, Japan, UK, US)^32^. In a unique admixed Brazilian cohort, genome-wide association study (GWAS) revealed both general and ancestry-specific genetic polymorphisms. Systems biology analysis revealed neuronal/synaptic signaling, monocyte count, glucose/lipid metabolism, and neurocognition/depression, as genetically linked to HAM/TSP patients, for which higher monocyte levels were validated in independent Brazilian and Peruvian cohorts^32^. Similar to our findings in HTLV-1-exposed A549 cells, Assone *et al*. found strong biological similarities between retroviral Hbz/Tax overexpression and HAM multi-omics findings, including viral pathways such as EBV, recently identified as the major driver of multiple sclerosis^32^. Finally, we compared our filtered KEGG gene list (Fig. 2f) with single-cell datasets of lung tissues from the Human Lung Cell Atlas^49^, which revealed a specific CCL2-high myeloid cell subset (Fig. 5i). Notably, this subset was strongly correlated with the previously defined HAM/TSP type I IFN gene signature^34^, indicating that these cells may contribute to the inflammatory responses observed in HTLV-1-exposed A549 cells.

The present study has two major limitations. First, confirming infection of A549 cells exposed to HTLV-1–infected cells or their SN is technically challenging. HTLV-1 primarily spreads via cell-to-cell contact through virological synapses, biofilm-like structures and cellular conduits, as well as through tunneling nanotubes^76,77^. Although the coculture model faithfully recapitulates HTLV-1 infection *in vitro*, it produces a complex mixture of donor and target cells, which complicates downstream analyses. While HTLV-1 infection in the lung is rare, previous studies have demonstrated the expression of viral proteins in HTLV-1-exposed alveolar epithelial cells^17^, and recent *in vivo* work has shown infection of respiratory tissues in HTLV-1-infected macaques^78^. Interestingly, our DEG analysis revealed significant transcriptional changes in A549 cells exposed to MT-2 SN compared to A549 control or A549 cells exposed to MT-4 SN (Fig. 2b). Notably, A549 cells co-cultured with MT-2 cells or their SN shared a large proportion of DEGs, with over 70 of our 105 selected genes differentially expressed under both conditions (Fig. 2f). This overlap suggests that, although residual MT-2 cell carryover in co-culture cannot be fully excluded, the observed biological effects are largely driven by HTLV-1 components present in the SN and remain biologically meaningful. Despite the rarity of *in vitro* HTLV-1 infection in A549 cells and the potential presence of residual MT-2 cells, HTLV-1 exposure induced marked transcriptomic reprogramming in A549 cells, consistent with *in vivo* findings in lung tissues. The second limitation lies in the absence of *in vivo* or single-cell data from HTLV-1–infected lung tissues. HTLV-1 is still a highly neglected virus, with limiting access to clinically relevant samples. Obtaining such datasets is also technically challenging due to the invasive nature of lung biopsies and the difficulty of securing ethical approval, which hampers the establishment of large public biobanks. Recently, however, a study in chimeric HTLV-1–infected macaques confirmed HTLV-1 involvement in the respiratory system^78^. Notably, all macaques infected with HTLV-1A cloned with the *Orf I* of the HTLV-1C strain developed bronchiectasis within 10 months of infection. The authors reported elevated IL-6, CCL2, and IL-1β levels in the lung. In addition, bronchoalveolar lavage (BAL) samples from the HTLV-1A–infected subgroup showed increased IL-15 and IL-1β, which were associated with higher frequencies of classical and non-classical monocytes producing IL-10 in blood^78^, corroborating our *in vitro* and *in silico* findings. Together, these findings demonstrate *in vivo* HTLV-1 infection in the lung and its strong association with bronchiectasis development in HAPD.

Despite these limitations, the main strength of this study lies in its multi-omics design. By integrating *in vitro* data and systems biology analysis, we were able to extend our findings to the *in vivo* level, combining multi-omics data from several independent cohorts worldwide, including healthy controls, people living with HTLV-1 and HAM/TSP patients.

## CONCLUSIONS

Our data-driven approach uncovers novel disease mechanisms and therapeutic targets for HTLV-1-associated lung pathology. Systems biology analysis showed RELA/ NF-κB p65 as the major upstream transcription factor for lung-specific HTLV-1-upregulated genes. A central role for CSF-1-mediated recruitment and differentiation of monocytes was mechanistically linked to NF-κB activation, as demonstrated using a CRISPR/Cas9 A549 RELA knockout cell line. A strong molecular overlap to both HAM/TSP and IPF reveals shared immunopathogenic pathways between unrelated pathologies targeting the lung. Together, these experimental and transcriptomic data support a model in which HTLV-1 drives chronic alveolar inflammation via epithelial-derived cytokine release and monocyte recruitment, while subsequent differentiation into inflammatory/profibrotic macrophages may contribute to viral persistence, immune dysregulation, and progression toward fibrotic lung disease. The *in vivo* relevance of our *in vitro* model was confirmed by integrated multi-cohort multi-omics analysis, combining bulk and single-cell transcriptomics, viral interactome and cross-ancestry GWAS.

## METHODS

### METHOD DETAILS

#### 1. Reagents

Recombinant human interleukin-1 beta (IL-1β) (#200–01B) and recombinant human CCL2 (#300–04) were purchased from PeproTech (Cranbury, NJ, USA). Recombinant Human Macrophage Colony-stimulating Factor (M-CSF) protein (#216-MC-010) was obtained from R&D Systems (Minneapolis, MN, USA). Mitomycin C (#A11491) was acquired from Adooq Bioscience (Irivine, CA; USA).

#### 2. Plasmids

pLentiCRISPRv2 plasmid was a gift from Feng Zhang (Addgene, Watertown, MA, USA, #52961). pCMV-VSV-G was a gift from Bob Weinberg (Addgene, #8454). LentiCRISPRv2 constructs were made based on the Zhang Laboratory protocol, using Quick ligase (New England Biolabs, #M2200S). pLV-SmCherry control plasmid was a gift from Pantelis Tsoulfas (Addgene, #36084). Concerning the design of shRNA cell lines, pLKO.1 - TRC cloning vector was a gift from David Root (Addgene, #10878). psPAX2 (Addgene, #12260) and pMD2.G (Addgene, #12259) were gifts from Didier Trono.

#### 3. Cell cultures

##### 3.1. Cell lines

Human lung carcinoma A549 (#CCL-185), Jurkat (Cat. No. TIB-152) and THP-1 cells (Cat. No. TIB-202) were purchased from American Type Culture Collection (ATCC, Manassas, VA, USA). HEK293T cells were received from Prof. Jason Moffat (Donnelly Centre, University of Toronto, Toronto, ON, Canada). MT-2 (active HTLV-1 producing cells) (#ARP237) and MT-4 (latently infected with HTLV-1) (#ARP120) cells were purchased from the National Institutes of Health (NIH) HIV Reagent Program.

A549 cells were maintained in HAM’s F-12K medium (Thermo Fisher Scientific [TFS], Waltham, MA, USA) supplemented with 5% fetal bovine serum (FBS, Cytiva, Marlborough, MA, USA) and 2mM L-Glutamine (TFS). HEK293T cells were grown in DMEM supplemented with 10% FBS and 2mM L-Glutamine (TFS). Jurkat, THP-1, MT-2 and MT-4 cells were cultured in RPMI (TFS) supplemented with 10% FBS (Cytiva) and 2mM L-Glutamine (TFS).

##### 3.2. Isolation and purity assessment of monocytes

Monocytes were isolated from buffy coats of healthy donors (Red Cross, Mechelen, Belgium; contract No. RKOV_19006) with informed consent. Erythrocytes were removed using HetaSep (STEMCELL, #07906) and human peripheral blood mononuclear cells (PBMCs) were obtained via density gradient centrifugation over Lymphoprep (STEMCELL Technologies, Vancouver, Canada, #18061). PBMCs were rotated overnight at 4°C to promote monocyte aggregations. Monocyte isolation was performed using the EasySep Human Monocyte Isolation Kit (STEMCELL Technologies, #19359) according to the manufacturer’s protocol. PBMCs (2×10⁸ cells in 2 mL EasySep Buffer) were incubated with 100 μL each of Isolation Cocktail and Platelet Removal Cocktail for 5 min at room temperature (RT), followed by addition of 100 μL of Magnetic Beads and an additional 5 min incubation. The volume was adjusted to 2.5 mL, and negative selection was performed using the EasySep Magnet to collect untouched CD14+ monocytes.

For purity assessment, PBMCs and isolated monocytes were washed and resuspended in PBS with 2% FBS at 10×10⁶ cells/mL. Human BD Fc Block (BD Biosciences, #564220) was added (25 μg per sample), and cells were incubated for 20 min at RT. Cells were then stained at 2×10⁵ cells/mL in 100 μL PBS + 2% FBS with 2.5 μL of each selected antibody. Staining was performed using anti-human CD3 APC-Cy7 (R&D System, #557832) and anti-human CD14 BV421 (R&D System, #563743), both from BD Biosciences. After 1 h at 4°C, cells were washed with PBS + 2% FBS and fixed in 200 μL PBS + 2% PFA.

##### 3.3. Genome editing

A CRISPR/Cas9-mediated RELA/NF-kB p65 knockout pool A549 cell line was generated using designed sgRNA sequences (Key Resources Table). Guide sequences were cloned into the pLentiCRISPRv2 plasmid (Addgene, #52961), according to the standard cloning protocol. For lentiviral particle production, HEK293T cells were plated in 40 mL supplemented DMEM in T150 (TPP, Trasadingen, Switzerland) flasks at 45% confluency and incubated overnight. One hour prior to transfection using the Lipofectamine LTX and Plus Reagent (TFS, #15338100), DMEM medium was removed and 13 ml OptiMEM^®^ (TFS, #31985062) was added to the flasks. The transfection mix was made by diluting 200 μl of PlusTM Reagent (TFS, #15338100) in 4 ml of OptiMEM^®^, in addition to 20 μg transfer plasmid (either lentiCRISPR v2 containing the sgRNAs, or pLV-mCherry), 10 μg of envelope vector pCMV-VSV-G (env gene) and 15 μg of packaging vector psPAX2 (gag, pol, rev and tat genes). In addition, 100 μl of lipofectamine LTX (TFS, #15338100) was diluted in 4 ml OptiMEM and added to the DNA and PlusReagent mix after 5 min. After 20 min of incubation at RT, the mixture was added in a dropwise manner to the T-150 flask HEK293T cells in OptiMEM. Six hours after transfection, the medium was removed and replaced with 30 ml DMEM containing 1% BSA. The supernatant containing lentiviral particles was harvested 60 h after transfection and stored at −80°C. A549 target cells were transduced with lentiviruses expressing a pool of the 2 sgRNAs and then selected with puromycin (1.5 mg/mL) for 3 days. A similar approach was followed to generate a MT-2 Tax shRNA cell line. Of note, packaging of shRNA lentiviruses was performed using psPAX2 and pMD2.G as envelop plasmids.

#### 4. Bulk RNA sequencing

##### 4.1. Sample preparation

A549 cells were seeded at 4×10⁵ cells per well in 6-well plates 24 h prior to infection or stimulation with cell culture supernatant (SN). On day 1, Jurkat, MT-4, and MT-2 cells were resuspended in RPMI at 4×10⁵ cells per mL and treated with 5 μM mitomycin C (Adooq Bioscience,#A11491) for 20 min at 37 C. Cells were washed with HAM’s F-12K medium and resuspended in the same medium at 4×10⁵ cells per mL. Finally, Jurkat, MT-4 or MT-2 cells were co-cultured with A549 cells at a final ratio of 1:1 (A549:Jurkat, MT-4 or MT-2). In parallel, SN from MT-4 and MT-2 cultures (collected 3 days post-passage) were filtered through a 0.45 μm filter (Corning, #431220) and used to stimulate A549 cells (mixed with control medium at a 1:1 ratio). After 24 h at 37 C, A549 cells were washed with PBS to remove non-adherent cells, detached with 0.25% trypsin, and incubated with CD25 Dynabeads (Invitrogen, TFS, #11157D) for negative isolation of A549 cells, according to the manufacturer’s instructions. RNA was then extracted using the RNeasy Mini Kit (Qiagen, Venlo, the Netherlands #74104) and the samples were submitted to the Genomics core facility (KU Leuven, Belgium) for RNA sequencing analysis (Supplementary Table 1).

##### 4.2. Principal Component Analysis

Principal Component Analysis (PCA) was performed to reduce the dimensionality of the dataset and to identify patterns in the multivariate data. The analysis was conducted using the prcomp function in R (v4.4.2).

##### 4.3. CIBERSORTx Deconvolution Analysis

To assess potential contamination of A549 transcriptomes with residual HTLV-1-infected donor cell material (MT-2 or MT-4), digital cytometry was performed using CIBERSORTx^31^. Normalized RNA-seq counts obtained from the different A549 co-cultures were input in CIBERSORTx for deconvolution. A custom signature matrix was generated from bulk RNA-seq profiles of MT-2 and MT-4 cells, derived from the same variants used in our *in silico* omics study (Vanderlinden et al., unpublished data). A549 monoculture from our RNA-seq analysis was used to define the epithelial cell profile in the signature matrix. The analysis was run in absolute mode with 100 permutations to estimate the relative abundance of MT-2 and MT-4–derived transcripts in each A549 sample. The resulting cell fraction estimates were statistically compared across experimental conditions using one-way ANOVA, followed by Dunnett’s multiple comparisons test to evaluate significant increases in donor cell-associated transcript signatures relative to controls. All values in both signature and mixture matrixes were presented as log(2) values.

##### 4.4. Differential gene expression analysis

To evaluate the impact of HTLV-1 infection on the A549 transcriptome, differential gene expression analysis was performed on various A549 co-cultures (Supplementary Tables 2–6). In this model, the A549-Jurkat co-culture transcriptome served as control to identify potential gene expression changes. Fold changes were also compared to established alveolar lung epithelial cell markers from single-cell data to focus on A549-specific effects (see 4.7). Raw reads were quality-checked with FastQC^31^ (v0.11.7), adapters trimmed using Trimmomatic^79^ (v0.39) and aligned to the **hg38** genome and transcriptome using hisat^80^ with default settings. Gene counts were obtained via FeatureCounts (Subread package^81^), and differential expression analysis was done with DESeq2^82^ in R software (v4.4.2). P-values were adjusted using the Benjamini-Hochberg method to control FDR. Simultaneously, all obtained mRNA reads were realigned and mapped to the reference HTLV-1 genome (**J02029.1**) to detect viral reads within the total RNA-seq data.

##### 4.5. KEGG and GO enrichment analyses

KEGG enrichment^83^ and Gene Ontology^84^ (GO) analyses were performed on the identified differentially expressed genes using the **clusterProfiler**^85^**, org.Hs.eg.db** (v3.19.0), **enrichplot** (v1.28.4), and **ggplot2**^86^ packages in R software (v4.4.2). Bar plots displaying the 40 most significantly enriched pathways for upregulated genes in A549 cells co-cultured with MT-2 cells were generated. From these, 18 pathways were selected based on their clinical relevance regarding HTLV-1 infection, to derive a filtered gene list of 105 upregulated genes in A549-MT-2 co-cultures for downstream protein-level analysis. Focus was given to pathways associated with oncogenic, neuroinflammatory, and respiratory viral infections.

In parallel, dot plots of significantly enriched GO terms were created to visualize enrichment results across five Gene Ontology (GO) categories: (1) **viral infection,** (2) **inflammation,** (3) **NF-κB activation,** (4) **cell chemotaxis,** and (5) **cell differentiation.**

##### 4.6. Protein-protein interaction network

A Protein–protein interaction (PPI) network was generated using the filtered list of 105 upregulated genes identified from A549–MT-2 co-culture transcriptomic data. Interactions were retrieved from the STRING database^87^ (v11.5) with a maximum confidence score of 0.9. The resulting PPI network was used to investigate signaling pathways and potential functional associations among the identified proteins. Particular attention was given to pathways directly linked to HTLV-1 infection (**hsa05166**), as well as those connecting infection to clinical outcomes such as bronchiectasis (**HP:0002110**) and increased levels of tissue monocytes (**BTO:0008876**). Additionally, the analysis emphasized monocyte responses within the pulmonary microenvironment by assessing enrichment in Gene Ontology (GO) terms related to monocyte chemotaxis (**GO:0002548**) and differentiation (**GO:0045655**). All selected pathways were significantly enriched, with a padj <0.05, after stringent FDR correction.

##### 4.7. Upstream Transcription Factor enrichment analysis

Prior to any *in vitro* experiments, an upstream transcription factor (TFs) enrichment analysis was performed to identify TFs likely to regulate the 105 pre-selected genes. This analysis was carried out using the open-source platform Enrichr^88^. Multiple databases documenting TF activity across diverse gene sets were assessed, with the ENCODE^89^ and TRUSTT^90^ databases ultimately chosen for the final analysis (Supplementary Table 7, Supplementary Table 8). In parallel, the filtered KEGG list of 105 genes was compared against the ARCHS4 Tissue database^91^ to determine the most likely tissue targets associated with enriched TFs.

##### 4.8. Cohort Presentation and Data Collection

Transcriptomics data (see 4.4) were first compared with publicly available transcriptomes from whole blood samples^34^. Subsequently, the results were contrasted with recent findings from multi-ancestry Genome-Wide Association Study (GWAS) data reported in a recent preprint^32^. Finally, both the transcriptomics and GWAS data were compared with an additional *ex vivo* HAM/TSP dataset (UCSF cohort)^36,37^, as well as with a curated Idiopathic Pulmonary Fibrosis (IPF) gene list, to evaluate potential links between HTLV-1–induced inflammation and clinical outcomes. All cohorts are summarized in Table 1 and described in detail in the referenced publications^32,34,36,37^.

Our study relied on publicly available data from the Gene Expression Omnibus (GEO), ensuring no ethical concerns or conflicts of interest. All patient data in the GEO datasets were previously collected under ethical approval and can be freely accessed and analyzed in accordance with GEO’s usage policies.

###### Epithelial cell status:

To validate the epithelial identity of A549 cells across co-culture conditions, RNA-seq–identified genes were compared against a curated list of epithelial cell markers sourced from the Panglao database^92^. Additionally, a correlation analysis was conducted by comparing raw gene counts from our RNA-seq data with aggregated read counts from multiple A549 RNA-seq experiments available in the ARCHS4 database^91^. Prior to comparison, the data were filtered to retain genes with a minimum of 200 reads in at least three samples to only look at commonly expressed genes in the defined cell line. Genes common to both datasets were then used to compute Pearson correlation coefficients, and a correlation heatmap was generated to assess sample similarity.

###### Idiopathic Pulmonary fibrosis (IPF):

To pinpoint IPF-related markers among differentially expressed genes (DEGs), a filtered list of IPF-associated genes was compiled from 5 GEO Series Matrix Files (**GSE32537**^40^**, GSE47460**^41–45^**, GSE53845**^46^**, GSE70866**^47^, and **GSE110147**^48^), including healthy and IPF patient samples. Expression data were normalized and analyzed with the **limma** package^93^ (v.4.4.2).

###### HTLV-1 gene signature in the Human Lung Cell Atlas:

To contextualize the results in a clinical perspective, the gene list derived from KEGG enrichment analysis was further examined using the Human Lung Cell Atlas database^49^, accessed via CellxGene Census. Relative gene expression levels were assessed using lung single-cell RNA-seq data. The HTLV-1 gene signature was compared across both healthy lung tissues and tissues affected by inflammatory lung diseases, including idiopathic pulmonary fibrosis (IPF), COVID-19, hypersensitivity pneumonitis, and pulmonary sarcoidosis. Furthermore, CCL2 expression in the lung was analyzed alongside ISG15 and CXCL10 within a defined myeloid cell subset.

#### 5. RT-qPCR

A549 cells were seeded at 4×10^5^ cells per well in 6-well plates 24 hours before infection or stimulation with cell culture SN. Unless specified differently, A549 cells were co-cultured with mitomycin-treated Jurkat, MT-4, or MT-2 cells (or SN) at a 1:1 ratio for 48 h. For the kinetics experiments, co-cultures were incubated for 6 h, 24 h, 48 h and 72 h. Then, the A549 cells were washed, and total RNA was extracted using the RNeasy Kit (Qiagen, #74104), according to the manufacturer’s instruction. First-strand cDNA was synthesized from 350 ng RNA using the High-Capacity cDNA Reverse Transcription Kit (Applied Biosystems, #4368814) and 10-fold diluted. Then, qPCR was performed with GoTaq qPCR Master Mix (Promega, Madison, WI, USA; #A6002) to quantify changes in mRNA expression levels. All primers (Key resources Table) were used at a final concentration of 500 nM. Amplification was performed on a QuantStudio 5 Real-Time PCR System (TFS), and consisted of a 2-min initial activation at 95°C, followed by 40 thermal cycles of 15 s at 95°C and 60 s at 60°C. A dissociation profile was taken at the end to confirm the specificity of the PCR amplification. Relative changes in gene expression were determined using the DDC_t_ values obtained for all tested primer pairs and normalized to either human GAPDH or b-globin as housekeeping genes. Ct values below detection threshold were ultimately defined as C_t_ = 35.

#### 6. Sandwich immuno-sorbent assay (ELISA).

Human CCL2 was analysed in cell culture supernatants from A549 control and A549 co-cultures, using the Human CCL2/MCP-1 (R&D Systems, #DCP00) ELISA kit, according to the manufacturer’s instructions.

#### 7. THP-1 migration assay

Chemotaxis experiments were performed, using a MultiScreen 96-well plate (Millipore, Burlington, MA, USA, #MAMIC5S10), as described before^94^. THP-1 cell migration through the 96-well filter plate occurs in response to a chemotactic gradient. First, the bottom side of the plate was filled with 150 ml of CCL2 (1–30 ng/ml; positive control) diluted in chemotaxis buffer (RPMI without phenol red and L-glutamine, supplemented with 0.1% bovine serum albumin), or with supernatants collected from A549 MT-2 co-cultures at different time points or at varying cell ratios. After placing the 96-well filter plate (5 mm pore size) on top, 100 μl of THP-1 cells at a concentration of 3.5×10^6^ cells per ml were seeded into the upper chamber. After a 3 h incubation at 37°C, the filter plate was carefully removed and discarded. Migrated THP-1 cells in the bottom plate were quantified using the luminescence ATP detection assay system (Revvity, #6016943). The bottom plate was centrifuged at 1200 × g for 5 min. Then, 50 μl of solution was carefully removed from the bottom plate and replaced with 50 μl of lysis buffer (at RT). The resulting 150 μl mixture (chemokine solution and lysis buffer) was transferred to a “view white” plate (Revvity, Waltham, MA, USA), and the plate was incubated on a shaker for 5 min at 400 × g. After adding 50 μl substrate solution (ATPlite, Revvity, #6016943) and shaking the plate again for 5 min at 400 × g, the plate was incubated in the dark at RT for 10 min, and reading of emitted luminescence was performed using ClarioStar Plus (BMG LabTech).

A chemotaxis index (CI) was calculated by dividing the luminescence value of the test sample by the luminescence value of the control buffer (n = 9 in 3 independent biological replicates per condition, padj < 0.05). Obtained CI were normalized to the A549 control condition and represented as log2-transformed values (Mean ± SEM).

#### 8. Differentiation of THP-1 cells and primary monocytes

To evaluate the effects of medium-derived chemokines on monocyte differentiation, THP-1 cells were cultured at 2×10^5^ cells per well in 6-well plates, either in RPMI control medium and conditioned medium from HTLV-1-infected (MT-2 SN) or non-infected (Jurkat SN) cells (collected 3 days after passage). All cultures were performed in a 1:1 ratio (i.e. 1 mL control medium + 1 mL conditioned cell culture SN). Cultures were maintained for 5 days, monitoring cell viability and confluency. Afterwards, THP-1 phenotype was examined microscopically, and macrophage markers were measured by RT-qPCR, following the method described above (Key resources Table).

Similar experiments were performed on purified monocytes, seeded at 1×10^6^ cells per well in 6-well plates, to verify the effects of cell culture-conditioned medium on primary cell differentiation.

### QUANTIFICATION AND STATISTICAL ANALYSIS

Statistical analyses were performed with Graphpad Prism (v9.5.1), whereas visualization of data was made in R software (v4.4.2). For RT-qPCR experiments, One-way ANOVA tests were performed with the assumption that the data followed a normal distribution. In case of high discrepancy between replicates, a non-parametric Kruskal-Wallis ANOVA was performed instead. Correlation analysis were performed using the Spearman correlation approach. An adjusted p value < 0.05 was the criterion for statistical significance. * = p < 0.05; ** = p < 0.01; *** = p < 0.001; **** = p < 0.0001. The tests used for each individual plot were mentioned in the figure legends.

## Supplementary Material

This is a list of supplementary files associated with this preprint. Click to download.

- SupplementaryFiguresfinal.pdf
- SupplementaryTables.xlsx

## Figures and Tables

**Figure 6:**
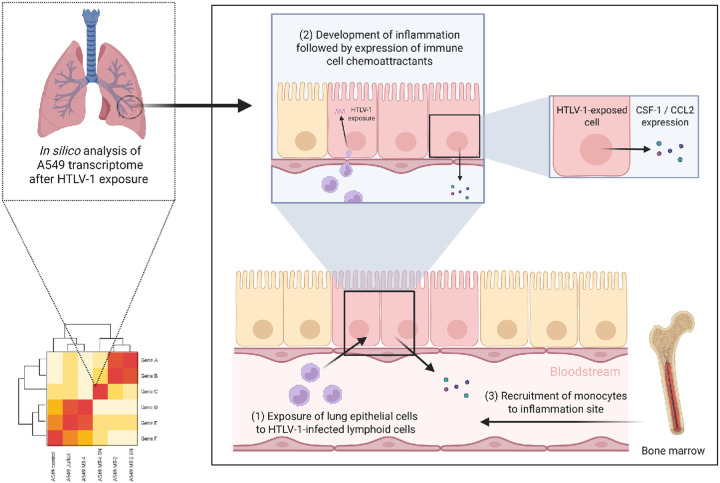
Legend not included with this version

**Table 1.** Summary of Cohorts Included in the Omics Analysis

Datasets used for Idiopathic Pulmonary Fibrosis (IPF) gene list				
Cohort	Number Control patients	Number IPF Patients	Sample Type	Omics Platform
GSE32537	50	167	RNA (lung)	MicroArray
GSE47460	108	254	RNA (lung)	MicroArray
GSE53845	8	40	RNA (lung)	MicroArray
GSE70866	20	212	RNA (lung)	MicroArray
GSE110147	11	22	RNA (lung)	MicroArray
Whole blood HTLV-1 transcriptomic signature				
Cohort	Number Control patients	Number AS/HAM patients^a^	Sample Type	Omics Platform
GSE29312	9	20/10	RNA (Blood)	MicroArray
Cohort Genome-wide associated study				
Cohort	Number AS patients^a^	Number HAM patients^a^	Sample Type	Omics Platform
Brazil	535	416	DNA	SNP Array
UCSF Cohort (nCounter)				
Cohort	Number Control patients	Number AS/HAM patients^1^	Sample Type	Omics Platform
UCSF	4	4/4	Blood	nCounter

a AS = Asymptomatic; HAM = HTLV-1-associated myelopathy/tropical spastic paraparesis.

**Table 2.** Alignment of obtained RNA sequencing reads to reference HTLV-1 genome

HTLV-1 gene	mRNA reads^a^				
	A549 Jurkat	A549 MT-4	A549 MT-4 SN	A549 MT-2	A549 MT-2 SN
Gag	0	0.2	0	2.2	0.6
Pro	0	0	0	0.2	0.2
Pol	0.2	0.7	0.3	22.8	4.2
Rex	0	0	0	0	0
Tax	0	0	0	0	0
Env	0	0	0	0	0
Hbz	0	0	0	0.6	0

a Average of mRNA reads obtained from the different A549 co-cultures mapped to an annotated HTLV-1 reference genome (n= 4–5 biological replicates per condition)

**Table 3.** Differential gene expression analysis

Condition	DEGs^a^	Upregulated DEGs^b^	Downregulated DEGs^b^
A549 Jurkat vs A549 control	0.7% (103/13742)	16% (16/103)	85% (87/103)
A549 MT-2 vs A549 Jurkat	8.5% (1304/15286)	69% (905/1304)	31% (399/1304)
A549 MT-2 SN vs A549 Jurkat	20% (2956/14900)	54% (1604/2956)	46% (1352/2956)
A549 MT-4 vs A549 Jurkat	0.6% (90/15673)	93% (84/90)	6.7% (6/90)
A549 MT-4 SN vs A549 Jurkat	0.6% (80/12586)	31% (25/80)	69% (55/80)

a Percentage of significantly differentially expressed genes (DEGs) in the different A549 co-cultures (padj <0.5). Data was normalized to the total number of transcripts, and A549 Jurkat co-culture was used as reference (except for A549 Jurkat, where A549 was used as reference).

b Percentage of significantly upregulated or downregulated genes within the statistically deregulated genes measured in the different A549 co-cultures.

**Table 4.** Supernatant of A549-MT-2 co-culture induces chemotaxis of THP-1 cells

Chemokine/Condition	Concentration (ng/ml)^a^	Chemotaxis Index^b^
CCL2	1.0	1.4 ± 0.3 ^ns^
	3.0	2.4 ± 1.4 ^ns^
	30	4.1 ± 2.5*
A549 control (6h)	3.8	1.0
A549 MT-2 (6h)	9.7	16 ± 0.7 ****
A549 MT-2 (24h)	64	11 ± 0.6****
A549 MT-2 (48h)	85	5.8 ± 0.7 ***
A549 MT-2 (72h)	73	3.8 ± 0.5*
A549 control (Cell ratio)	18	1.0
A549 MT-2 (4:1)	60	6.3 ± 1.0
A549 MT-2 (2:1)	76	8.7 ± 1.8**
A549 MT-2 (1:1)	85	4.8 ± 1.4 ^ns^
A549 MT-2 (3:5)	97	5.3 ± 1.7 ^ns^
A549 MT-2 (1:2)	86	4.2 ± 1.4 ^ns^

a CCL2 concentration present in the different cell culture supernatants was measured by ELISA (n = 3 biological replicates per condition).

b Chemotaxis Index was calculated by dividing the luminescence value of the test sample by the luminescence value of the control buffer. Obtained CI were normalized to the A549 control condition and represented as on graphs as log2-transformed values (Mean ± SEM). (n = 9 in 3 independent biological replicates per condition, padj < 0.05). Statistical analysis by one-way ANOVA with Tukey post hoc test;

* p < 0.05,

** p < 0.01,

*** p < 0.001,

**** p < 0.0001.

**Table:** KEY RESOURCES TABLE

REAGENT OR RESOURCE	SOURCE	IDENTIFIER
**Antibodies**		
Mouse anti-Human Clathrin	BD Biosciences	610500 (Western blot)
NF-kB p65	R&D Systems	MAB5078 (Western blot)
Goat Anti-Mouse HRP	Agilent Dako	P0447 (Western blot)
Fc Block (Flow cytometry)	BD Biosciences	564220 (Flow cytometry)
Mouse anti-Human CD3 APC-Cy7	R&D Systems	557832 (Flow cytometry)
Mouse anti-Human CD14 BV421	R&D Systems	563743 (Flow cytometry)
Mouse anti-Human CD54 PE	BD Biosciences	347977 (Flow cytometry)
**Bacterial and virus strains**		
NEB 10-beta/Stable Competent *E.coli*	New England Biolabs	C3040H
**Biological samples**		
Buffy coats (Healthy donors)	Red Cross, Mechelen, Belgium	RKOV_19006
**Chemicals, peptides, and recombinant proteins**		
Recombinant Human Interleukin IL-1b	PeproTech	200–01B
Recombinant Human CCL2	PeproTech	3000–04
Recombinant Human Macrophage Colony-stimulating Factor	R&D Systems	216-MC-010
**Critical commercial assays**		
RNeasy Kit	Qiagen	74104
AllPrep DNA/RNA/Protein Kit	Qiagen	80004
High-Capacity cDNA Rever Transcription Kit	Applied Biosystems	4368814
GoTaq qPCR Master Mix	Promega	A6002
ATPlite Luminescence Assay System 96-well	Revvity	6016943
Human CCL2/MCP-1 ELISA kit	R&D Systems	DCP00
EasySep Human Monocyte Isolation Kit	STEMCELL Technologies	19359
Quick Ligation Kit	New England Biolabs	M2200S
**Deposited data**		
Transcriptomics data generated in this study are currently under submission.		
**Experimental models: Cell lines**		
MT-2	NIH HIV Reagent Program	ARP237 (Engineered)
MT-2 Tax shRNA	NIH HIV Reagent Program	ARP237
MT-4	NIH HIV Reagent Program	ARP120
Jurkat	ATCC	TIB-152
THP-1	ATCC	TIB-202
A549	ATCC	CCL-185
A549 NF-kB p65 KO	ATCC	CCL-185 (Engineered)
HEK293T WT	ATCC	CRL-3216
**Experimental models: Organisms/strains**		

**Oligonucleotides**		
**Name**	**Sense strand**	**Antisense strand**
CRISPR/Cas9 NF-kB p65 KO Exon 6	ACTACGACCTGAATGCTGTG	CACAGCATTCAGGTCGTAGT
HTLV-1 Tax shRNA knockdown	GCAGATGACAATGACCATGA	TCATGGTCATTGTCATCTGC
GAPDH	TGATTTTGGAGGGATCTCGCTCCTGGAA	GTGAAGGTCGGAGTCAACGGATTTGGTCGT
b-Globin	GCAAGAAAGTGCTCGGTG	CTACTCAGTGTGGCAAAGGTG
HTLV-1 Tax	CTACATCGTCACGCCCTACT	ATGAGTGATTGGCGGGGTAA
HTLV-1 Hbz	AGAACGCGACTCAACCGG	TGACACAGGCAAGCATCG
IL-1b	AGATGATAAGCCCACTCTACAG	ACATTCAGCACAGGACTCTC
TNF-a	CCCGAGTGACAAGCCTGTAG	GATGGCAGAGAGGAGGTTGAC
IL-6	ACAGCCACTCACCTCTTCAG	CCATCTTTTTCAGCCATCTTT
CXCL8	AGACAGCAGAGCACACAAGC	ATGGTTCCTTCCGGTGGT
CSF-1	GTTTGTAGACCAGGAACAGTTGAA	CGCATGGTGTCCTCCATTAT
CSF-1R	GCTGCCTTACAACGAGAAGTGG	CATCCTCCTTGCCCAGACCAAA
IL-34	AATCCGTGTTGTCCCTCTTG	CAGCAGGAGCAGTACAGCAG
CCL2	GCCCCAGTCACCTGCTGTTAT	CTGCTTGGGGTCAGCACAGA
CD11b	CAGCCTTTGACCTTATGTCATGG	CCTGTGCTGTAGTCGCACT
CD14	AGCCAAGGCAGTTTGAGTCC	TAAAGGACTGCCAGCCAAGC
CD16	ATGTGTCTTCAGAGACTGTGAAC	TTTATGGTCCTTCCAGTCTCTTG
CD36	GCCAAGGAAAATGTAACCCAGG	GCCTCTGTTCCAACTGATAGTGA
CD68	GCTACATGGCGGTGGAGTACAA	ATGATGAGAGGCAGCAAGATGG
CD86	CTGCTCATCTATACACGGTTACC	GGAAACGTCGTACAGTTCTGTG
CD163	CAGGAAACCAGTCCCAAACA	AGCGACCTCCTCCATTTACC
CD169	CCTCGGGGAGGAACATCCTT	AGGCGTACCCCATCCTTGA
CD206	TTCGGACACCCATCGGAATTT	CACAAGCGCTGCGTGGAT
**Recombinant DNA**		
pPLentiCRISPRv2 plasmid	Addgene	52961
pCMV-VSV-G	Addgene	8454
pLV-SmCherry	Addgene	36084
pLKO.1	Addgene	10878
psPAX2	Addgene	12260
pMD2.G	Addgene	12259
**Software and algorithms**		
CLC Main WorkBench	Qiagen	v22.0.2
Enrichr	Icanh School of Medicine at Mount Sinai (Ma’ayan Laboratory)	https://maayanlab.doud/Enrichr/cha
ShinyGO	South Dakota State University	v0.85
FlowJo	BD Biosciences	v10.8.1
GraphPad Prism	GraphPad Software	v10.6.0
Design and Analysis Software	ThermoFisher Scientific	v2.6.0
Image Lab	Bio-Rad	v6.1
R	The R Project for Statistical Computing	v4.4.2
RStudio	Posit PBC	V2024.09.01
STRING	Global Biodata Coalition and Elixir	v12.0
CELLxGENE Census	Chan Zuckerberg Initiative	CZ CELLxGENE Discover - Cellular Visualization Tool
CIBERSORTx	Stanford University	Newman *et al*. (2019).
**Others**		

## Data Availability

Transcriptomic data generated in this study will be made publicly available online (under submission). All materials generated in this study will be provided upon request.
